# Supplementary material for: Diagnostics to support the control of scabies–Development of two target product profiles
Source: PLoS Negl Trop Dis. 2022 Aug 30;16(8):e0010556. doi: 10.1371/journal.pntd.0010556 (PMC9467343; doi:10.1371/journal.pntd.0010556)
Supplement: Attachment — Submitted filename: 220716_TPP Revision.docx [file pntd.0010556.s003.docx]

Dear PLOS NTDs

Many thanks for provisionally accepting our manuscript for publication in PLOS NTDs and for the useful comments from the peer reviewers. We provide a point by point response below and have submitted a revised version of the manuscript addressing the comments.

Yours sincerely,

Dr Michael Marks, Clinical Research Department, London School of Hygiene & Tropical Medicine

**Editorial Comments**
I would suggest that you replace the letters with the numbers after the authors names and use these numbers (in superscript and adjacent to the first letter of the address) also for the addresses.

We have made this change.

Correct the sentence: i) establishing if the proposed % community prevalence of scabies for initiating MDA (10%) had been met (starting MDA)…

We have made this change.

Is there a reason to start with Table 2?> Give explanations what the * in table 2 (1) means

We have corrected this typo and renumbered the tables starting from 1 and replaced the * with an in-line explanation.

Table 3: Replace the sign ^ with a and continue with b,c,d, instead of ^c, d, and 3!! Why is 3 written in red? Give explanations what all these mean. Table 4: Give explanations for ^ and 3, replace them with a and b.
Is there any reason why the 3 (whatever it is) is placed in front of the percentages?

The 3s were typos carried over from an internal WHO document. This explanation was given under the table. We have replaced the ^ with * and provide the requested information under each table

Table 3 and 4: Write everywhere S. scabiei. Separate twice Sarcoptesscabiei and replace with S. scabiei (always in italics)

We have corrected this

Throughout the text leave a space before the reference numbers.

We have tried to do so but presume this will be adjusted at the copy-editing stage

 
Reviewer #2: Authors do a reasonable job describing the TPP process and the basic assumptions used to make the sensitivity and specificity calculations. They should describe the assumptions about the survey design (as cluster design will impact these calculations). It would be helpful to include a table that clearly lays out the various assumptions, as this would help the user understand better how changes in our understanding of the accuracy of the assumptions would impact the TPP

We have added in to the methods outlining a summary of the assumptions based on the WHO informal consultation on scabies

“These recommendations assume that survey will be conducted using cluster randomized, whole age household survey designs with decisions about MDA being made at the level of the whole evaluation unit. There is currently no agreed design effect for use in scabies surveys so for the purpose of the TPP development we used a design effect of 2.”

2. In the table about the diagnostic landscape (table 2): are the specificities and sensitivities for the different modalities data-based or expert opinion? Citations should be provided for those numbers that are evidenced-based. If there is a limited amount of evidence, a note to the table indicating that the numbers are expert opinion except when indicated would be important. Also indicate #s from the grey literature.

We have made explicit that for the existing tests estimates of sensitivity and specificity are based on expert opinion informed by the literature.

4. Very curious as to why the ideal test is LAB-based and not POC. This would be something that would be important to mention in the discussion, as it seems that it would be the other way around.

We are unclear why the reviewer believes the ideal test should be lab based as this is not stated in the manuscript. To avoid confusion we have made this more explicit in the results

Would expound a bit more about the World Scabies Program (and perhaps provide the link); despite its name it is quite small (and underfunded) but it is an important first step towards scale up. A sentence or two describing its structure and funding and clearly stating that it is not a WHO organization would be important.

We have made clear that WSP is in Australia and therefore not a WHO organization. The role of WSP falls outside the scope of this manuscript so we have not added beyond this .

2. The TPP seems to address the 3 problem challenged mentioned in the 1st paragraph of the discussion, not the second.

We are unclear that the specific point being made here is. The TPP is specifically designed to address problems mentioned in the first paragraph of the discussion as the reviewer notes.

I was looking for a discussion of how potential changes in the provision thresholds might impact the TPP. Is it likely that the thresholds will go up or down? Which would be more problematic for the TPP? What are the implications of not starting MDA (given the 20% risk of not detecting 10% prevalence) vs starting MDA when it not needed (given the 5% risk of finding >10% when the prevalence is 8%)?

The implications of not starting MDA and why this balance was chosen is mentioned in the discussion where we state.
“For this TPP, the expert subgroup considered the criteria needed to accurately start MDA at least 80% of the time when the prevalence of scabies was at least 10% and to not incorrectly start if the prevalence was 8% or less more than 5% of the time. The requirement to avoid initiation of MDA reflects, in part, the current absence of evidence about the effectiveness and cost-effectiveness of MDA as a strategy at lower prevalences alongside also the lack of a current drug donation to support MDA progammes, rather than an intrinsic biological rationale for the current threshold. Equally thresholds for stopping reflect expert consensus rather than empirically derived thresholds below which rebound of disease is unlikely following cessation of MDA.”

We would be happy to expand on this if there was a specific element the reviewer felt was missing but given the considerable uncertainty about the effectiveness and cost-effectiveness of MDA at different thresholds it is difficult to give precise information on this point.

I was also looking for a discussion about how the uncertainty around the calculation of sens/spec of non-expert clinical exam and how that impacts sens/spec calculations for the minimum requirements would be important; should the focus be on a test that does not require exam? Why or why not?

We have expanded upon this. Essentially if the sensitivity of initial clinical examination was substantially below 80% then the two-stage process becomes less viable as a strategy (because with lower sensitivity you do not pass enough individuals on to the second step of testing) in particular for the initiation of MDA. We have included this information in the discussion.

Numerous typos, run-on sentences, spacing errors, extra periods Genus/species names that are not italicized, etc. Authors should review thoroughly and correct. If future versions of the paper included line numbers it would be easier for review to provide specific feedback.

We have tried to correct typos

If any changes to the TPP were made based on public comment, it would be nice to have that pointed out.

No changes were made and we have added this information
